# Short-term cognitive effects of repeated-dose esketamine in adolescents with major depressive disorder and suicidal ideation: a randomized controlled trial

**DOI:** 10.1186/s13034-023-00647-2

**Published:** 2023-09-14

**Authors:** Xiaofeng Lan, Chengyu Wang, Fan Zhang, Haiyan Liu, Weicheng Li, Yanxiang Ye, Zhibo Hu, Siming Mai, Yuping Ning, Yanling Zhou

**Affiliations:** 1 Department of Child and Adolescent Psychiatry, Affiliated Brain Hospital of Guangzhou Medical University, Mingxin Road #36, Liwan District, Guangzhou, 510370 China; 2 Key Laboratory of Neurogenetics and Channelopathies of Guangdong Province and the Ministry of Education of China, Guangzhou Medical University, Guangzhou, 510370 China; 3 Guangdong Engineering Technology Research Center for Translational Medicine of Mental Disorders, Guangzhou, 510370 China; 4 Department of Psychology, The First School of Clinical Medicine, Southern Medical University, Guangzhou, 510515 China

**Keywords:** Esketamine, Suicidal ideation, Adolescent, Depression, Cognition

## Abstract

**Background:**

Ketamine and its enantiomer have rapid and robust effects on depressive symptom and suicidal ideation. Little is known about their cognitive effects in adolescents. We aimed to evaluate the short-term effect of esketamine on cognition in adolescents with major depressive disorder (MDD) and suicidal ideation.

**Method:**

In this randomized-controlled trial, 51 participants aged 13–18 with MDD and suicidal ideation received three intravenous infusions of either esketamine (0.25 mg/kg) or midazolam (0.02 mg/kg). Four dimensions of the MATRICS Consensus Cognitive Battery (MCCB), including processing speed, working memory, verbal learning and visual learning, were assessed at Days 0, 6 and 12.

**Results:**

In the linear mixed model, a significant time main effect (F = 12.803, P < 0.001), drug main effect (F = 6.607, P = 0.013), and interaction effect (F = 3.315, P = 0.041) was found in processing speed. Other dimensions including working memory and verbal learning showed significant time main effect (all P < 0.05), but no significant drug or interaction effect (all P > 0.05). Esketamine group showed improvement in processing speed from baseline to Days 6 and 12, and working memory from baseline to Day 12 (all P < 0.05). The generalized estimation equation showed no significant association between baseline cognition and antidepressant or antisuicidal effect (both P > 0.05).

**Conclusions:**

The present study suggested that three-dose subanesthetic esketamine infusions did not harm cognition among adolescents with MDD and suicidal ideation. Instead, esketamine may be associated with improvement in processing speed.

*Trial registration*: This trial was registered in the Chinese Clinical Trials Registry (http://www.chictr.org.cn, ChiCTR2000041232).

**Supplementary Information:**

The online version contains supplementary material available at 10.1186/s13034-023-00647-2.

## Introduction

Ketamine, an *N*-methyl-d-aspartate (NMDA) receptor antagonist, has been used for decades in the induction and maintenance of anesthesia. (R, S)-ketamine (ketamine) and its enantiomer, esketamine, have a rapid and robust effect on depressive symptom and suicidal ideation [1–7]. Recently, the intranasal esketamine (Spravato) was approved by the U.S. Food and Drug Administration for treatment-resistant depression (TRD) and major depressive disorder (MDD) with acute suicidal ideation or behavior in adults [3, 4].

Given the safety and success of ketamine and its enantiomer in treating depression in adult population, clinical studies aiming to evaluate their efficacy and safety in adolescents have been already started. Currently, the only randomized placebo-controlled trail of single low dose ketamine infusion in 17 adolescents with TRD suggested ketamine might be well tolerated and effective in reducing depressive symptom [8]. Two small open-label trials, one including 13 adolescent TRD and the other including 12 adolescents with treatment-refractory bipolar depression also suggested that ketamine was an effective and safe treatment for this adolescent population [9, 10]. In addition to the antidepressant efficacy highlighted above, three case reports indicated benefits of ketamine on suicidal ideation in adolescents [11–13]. These limited data did not identify any high-frequency or concerning serious adverse events and draw a preliminary conclusion that low-dose ketamine was well tolerated in adolescents.

Concerns about ketamine’s adverse reactions for adolescents will persist until larger studies can demonstrate it is safe enough for this population. One worry is that it would harm cognitive function, because recreational abuse of ketamine was related to cognitive deficits in executive functioning, episodic memory and working memory [14, 15]. Long-lasting cognitive impairments following chronic ketamine exposure subjects presented the worrisome possibility that even a therapeutic dose of ketamine would harm cognitive function in adolescents. Systematic examination of ketamine’s effects on cognitive function in the context of subanesthetic dose ketamine treatment in adolescents is warranted.

To date, no known clinical trial has systematically examined the effect of repeated intravenous (IV) ketamine or its enantiomer on cognition in adolescents, but some answers can be obtained from adult data. Results of adult studies have been inconsistent with some reporting no changes in cognition, including processing speed, working memory and visual learning [16–18], some a slight decrease in episodic memory and delay recall, verbal memory, semantic processing and sustained attention [19, 20] and others an improvement in cognition including in visual and verbal memory, processing speed, set shifting, and working memory [17, 21–23]. Our previous open-label study examined the cognitive effects of six-dose of IV ketamine over 2 weeks in unipolar and bipolar depression [18, 24]. We found simple improvements in verbal learning and processing speed at 24 h post-treatment, and the improvements in processing speed were partly independent of improvements in depressive symptoms [18, 24]. Long-term studies conducted in large samples also provided evidence for the safety of esketamine on cognition in adults. An open-label, multicenter, long-term study evaluated the safety of esketamine nasal spray in 779 adults with TRD, suggesting that cognitive performances, including measures of simple and choice reaction time, visual and verbal learning and memory, working memory, and executive function, either improved or remained stable from baseline through 44 weeks of maintained treatment phrase (weekly or every-other-week esketamine administration) [25]. Similar findings were reported by another long-term (up to 4.5 years) study of 1148 TRD patients treated with esketamine, indicating that cognitive performances, including attention, visual learning and memory, and executive function, the Hopkins Verbal Learning Test-Revised, generally remained stable from baseline through week 160 after the same maintained therapy phrase (weekly or every-other-week administration) [26]. Overall, the above-mentioned evidences indicated that there were no severe and persistent cognitive impairments in depressed adults treated with ketamine [27, 28].

Ketamine has different effects on cognitive performances of abusers and patients receiving therapied ketamine, which may be due to different doses and exposure duration. Cognitive deficits were mainly observed in frequent ketamine users and chronic ketamine abusers (mostly > 20 months) [14]. Average doses of ketamine use in abusers ranged from 2.77 to 3.5 g per day [14, 29] and frequency of use ranged from 6.0 to 6.5 days per week [29, 30]. However, the frequency of ketamine treatment in patients with depression was much lower, generally twice- or thrice-weekly infusion in a 2- week treatment period, with low doses ranging from 0.2 mg/kg to 0.75 mg/kg [17, 23]. That means that there might be no severe cognitive deficit when patients received subanesthetic doses of ketamine treatment in a low frequency. But it is unclear whether therapied esketamine infusions have similar effects on cognition of adolescent population with MDD.

Herein, we aim to evaluate the effects of esketamine on cognition in the context of a placebo-controlled prospective study in adolescent population with MDD and suicidal ideation that examined the efficacy and safety of three infusions of esketamine. Based on our previous findings from adults, we hypothesized that three low-dose infusions of esketamine administrated to adolescents would not be associated with a decline in cognition relative to baseline. Then, we explored whether the level of cognitive performance at baseline influenced the antidepressant and antisuicidal outcomes observed.

## Methods

### Study design

The data presented herein were obtained from patients referred to a randomized (1:1), double-blind, placebo-controlled trial which was conducted in the Affiliated Brain Hospital of Guangzhou Medical University between December 2020 to April 2022. This trial was approved by the Clinical Research Ethics Committees of the Affiliated Brain Hospital of Guangzhou Medical University, and registered in the Chinese Clinical Trials Registry (http://www.chictr.org.cn, ChiCTR2000041232). The trial protocol was published elsewhere in detail [31]. The primary purpose of this trial was to evaluate the safety and efficacy of three-dose esketamine compared to midazolam in patients with MDD and current suicidal ideation. Current study was a secondary analysis of existing trial data.

### Participants

Adolescents with major depressive episodes were recruited from the inpatient ward of the Affiliated Brain Hospital of Guangzhou Medical University. Inclusion criteria were: age 13–18 years, diagnosis of MDD without psychotic features according to DSM-5 criteria, the 17-item Hamilton Depression Rating Scale (HAMD-17) total score ≥ 17 at screening, and had current suicidal ideation for ≥ 3 months as measured by a clinician-rated Columbia suicide severity rating scale (C-SSRS) suicidal ideation score ≥ 1 and a self-report Beck Scale for Suicide Ideation (SSI) item 4 or 5 score ≥ 2 at screening. Exclusion criteria included the presence of substance use and alcohol addiction, primary psychotic, bipolar disorder, pervasive developmental, posttraumatic, obsessive–compulsive and nonpsychiatric neurological disorders, a significant medical illness, or active suicidal attempt on presentation or in the preceding 6 months. All participants were physically healthy according to the results of physical exam, blood laboratory testing and electrocardiogram.

### Randomization and blinding

Participants were randomized in a 1:1 ratio to receive three infusions of either esketamine hydrochloride or midazolam via a computer-generated randomization scheme. Midazolam was used as an active control, in keeping with its similar pharmacokinetic profile and precedent as a reasonable comparator for esketamine’s nonspecific behavioral effects. Study drugs were administered on Days 1, 3 and 5.

The raters and participants as well as their clinician were kept blind to the assigned treatment at randomization. A designated psychiatrist generated the random allocation sequence and assigned participants to interventions, but did not participate in enrollment participants and assessment scales. This psychiatrist prepared the study drug with a nurse who did not participate in other aspects of the study within each participating department. Both esketamine and midazolam perfusions were transparent and visually similar.

### Procedures

Participants fasted overnight prior to study drug administration, until two hours after the start of infusion. Esketamine 0.25 mg/kg or midazolam 0.02 mg/kg in 50 ml 0.9% of saline was infused over 40 min via an infusion pump. Each infusion was clinically monitored and regular measures of pulse and blood pressure were checked before infusion, 20 min during infusion, and at the end of infusion (about 40 min). A physician was present during all infusions and monitored the participant. The infusion would be discontinued when participants suffered any intolerable treatment-emergent status, including hemodynamic instability, severe dissociation, severe psychotic symptom, impulsive behavior, worsening depression, suicide and anxiety.

All potential participants were admitted to hospital and received standard care treatment. During infusion phase (Days 1–5), current oral medication treatment maintained. During the naturalistic follow-up phase (Days 6–12), participants were treated with necessary antidepressant treatment, managed by clinician. Structured psychotherapy, repetitive transcranial magnetic stimulation (TMS) and electroconvulsive therapy (ECT) were not allowed to take throughout the infusion phase. Additional details on the study procedures have been published in the trial protocol [31].

### Clinical symptom and cognitive function measurements

Clinical measures included depressive symptom and suicidal ideation. The severity of depressive symptom was measured via the Montgomery–Asberg Depression Rating Scale (MADRS) [32] at baseline, 1 day following the last infusion (Day 6), and again one week after the third infusion (Day 12). The total scores of MADRS range 0–60, with higher scores indicating more severe depression symptoms. Current suicidal ideation, rated by the self-report SSI, scaled 0 (least severe) to 2 (most severe) for each item [33]. The first 5 items of SSI (SSI-5): wish to live, wish to die, reasons for living/dying, desire to make a suicide attempt, and passive suicidal thoughts were rated at baseline, Days 6 and 12.

Cognitive function was assessed using the Chinese version of MATRICS consensus cognitive battery (MCCB) administrated at baseline, Days 6 and 12, which has been demonstrated to be a sensitive battery of cognitive impairment in young MDD patients [34]. Four dimensions were selected from the MCCB, including processing speed, working memory, visual learning and verbal learning. Processing speed was measured by Brief Assessment of Cognition in Schizophrenia (BACS), Trail Making A test, and the Category Fluency test. Working memory was evaluated using the subtests Spatial Span of the Wechsler Memory Scale-III. Visual learning and verbal learning were assessed by the Hopkins Verbal Learning Test-Revised, and the Brief Visuospatial Memory Test-Revised, respectively. There are three test versions, version A, B and C, in visual learning and verbal learning, which had different topics but equal difficulty. In order to reduce potential learning effect in subjects, we used version A at baseline, version B at Day 6 and version C at Day 12. The score of each dimension in MCCB was standardized to a T score with a mean of 50 and a standard deviation of 10, with higher score indicating better cognition. Although the MCCB is initially developed for the assessment of cognitive function in population with schizophrenia, it has also been widely used in individuals with affective disorders in recent years [35, 36]. The above four cognitive dimensions were chosen because memory and learning dysfunction often occur in depression [37], which were also measured by a range of clinical research using ketamine as a treatment drug [27, 28].

During the follow-up phase, the aforementioned evaluations of clinical symptoms and cognition were required to be completed even if the participants received only one or two infusions.

### Statistical analyses

Power analysis determined sample size using a meta-analytic estimate of effect size = 0.85 for the change in clinician-rated suicidal ideation score for a single dose of ketamine vs control [5]. This effect size requires a sample size of 22 participants per treatment group to achieve 80% power at two-sided significance level of 0.05. Assuming a 10% dropout rate after the first infusion, at least a sample size of 25 participants was needed for each treatment group.

All analyses were conducted in IBM SPSS Statistics version 27, with a significance level set to 0.05. Statistical analysis including participants who lacked a Day 12 visit was performed in accordance with the intention-to-treat (ITT) principle.

Demographic and clinical characteristics at baseline were compared between groups using the Student’s t-test for continuous variables and Chi-squared test for categorical variables.

Then, a mixed-effects repeated measures model was conducted to examined differences in MADRS score, SSI-5 score and each dimension of MCCB between groups from baseline to endpoint (Day 12). The model included time (baseline, Days 6 and 12), drug (esketamine and midazolam) and time-by-drug interaction as fixed factors, oral antidepressant class [selective serotonin reuptake inhibitor (SSRI) or non-SSRI], plus augmentation therapy (yes or no) and the respective baseline score for the measurement as covariates. Bonferroni-corrected simple effects post hoc tests were used to examine changes of each measurement from baseline to each follow-up point in each group as well as drug differences in timing. Effect sizes (Cohen’s d) were calculated using mean difference between two groups at each time point, and difference between baseline and follow-up point.

Finally, a generalized estimation equation was used to assess the influence of baseline cognitive performance on esketamine’s effect at Days 6 and 12. In this model, follow-up (Days 6 and 12) was within-subject variable, antidepressant response (defined as an improvement ≥ 50% from baseline in MADRS total score), remission of depressive symptoms (defined as a MADRS total score ≤ 12) and being free of suicidal ideation (defined as SSI-5 score of 0) was included as dependent variable respectively, and four dimensions of baseline MCCB score were included as independent variables, with study drug, oral antidepressant class and plus augmentation therapy as covariates. The similar generalized estimation equation was conducted in each subgroup (esketamine and midazolam) sample, while study drug was excluded from covariates.

## Results

### Participants characteristics

**Fig. 1:**
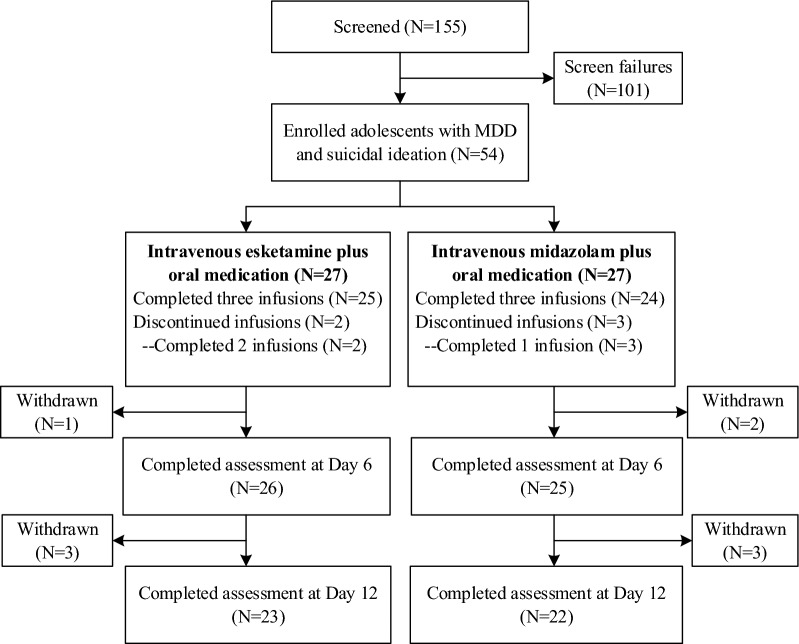
Flow chart of the patient inclusion process

From the original trial, a total of 155 participants were screened for eligibility, and 54 were enrolled and received at least one infusion of study drug, but 5 participants failed to complete all three infusions (3 in the midazolam group and 2 in the esketamine group). 51 participants (25 in the midazolam group and 26 in the esketamine group) who completed measurements of cognitive performance and clinical symptoms both at baseline and Day 6 were included in the final analyses. 88.2% of them (N = 45) completed the last follow-up assessment (Day 12). The flow chart of the study was presented in Fig. 1.

Demographics and clinical details of participants were shown in Table 1. The esketamine and midazolam groups were well matched in age, education, gender, body mass index (BMI), duration of illness, and current oral medication types. Baseline MADRS total score, SSI-5 score and four dimensions of MCCB did not differ significantly between groups.

**Table 1:** Demographic and baseline characteristics

	Midazolam (N = 25)		Esketamine (N = 26)		t	df	P
	Mean	SD	Mean	SD			
Age (years)	14.6	1.3	15.0	1.4	− 1.063	49	0.293
Education (years)	8.5	1.4	8.8	1.6	− 0.766	49	0.447
Duration of illness (months)	19.7	14.5	21.7	10.4	− 0.550	49	0.585
BMI (kg/m^2^)	19.5	4.1	21.1	3.3	− 1.524	49	0.134
MADRS score	36.1	7.4	35.5	6.5	0.298	49	0.767
SSI-5 score	8.6	1.5	8.1	1.4	1.396	49	0.169
Processing speed	42.2	11.6	41.2	8.8	0.364	49	0.717
Working memory	43.9	12.6	42.2	10.4	0.535	49	0.595
Verbal learning	43.2	11.6	46.7	9.9	− 1.134	49	0.262
Visual learning	42.9	10.3	44.6	7.4	− 0.661	49	0.512

	N	%	N	%	χ^2^	df	P
Female	23	92.0	22	84.6	0.670	1	0.668
Current nonsuicidal self-injury	25	100	26	100	–	–	–
Mood disorder in first degree relative	10	40.0	7	26.9	0.981	1	0.322
First in hospital	15	60.0	17	65.4	0.158	1	0.776
First episode	18	72.0	20	76.9	0.163	1	0.755
Current antidepressant	31	100	30	100	–	–	–
SSRIs	23	92.0	20	76.9	2.191	1	0.248
SNRIs	2	8.0	5	19.2	–^a^	–	0.419
Current antipsychotic	12	48.0	19	73.1	3.362	1	0.089
Current benzodiazepine	21	84.0	16	61.5	3.229	1	0.116

*BMI* Body mass index, *MADRS* Montgomery–Åsberg Depression Rating Scale, *SSI* Beck Scale for Suicide Ideation, *SSRIs* Selective serotonin reuptake inhibitors, *SNRIs* Serotonin norepinephrine reuptake inhibitor

^a^Fisher’s exact test

### Clinical outcomes

Three infusions of esketamine were significantly associated with reductions in MADRS total score and SSI-5 score, which have been reported detailedly elsewhere [45]. The proportions of the antidepressant responders and remitters as well as being free of suicidal ideation at Day 12 were 20.0%, 8.0% and 16.0% in midazolam group, and 38.5%, 26.9% and 30.8% in esketamine group.

**Fig. 2:**
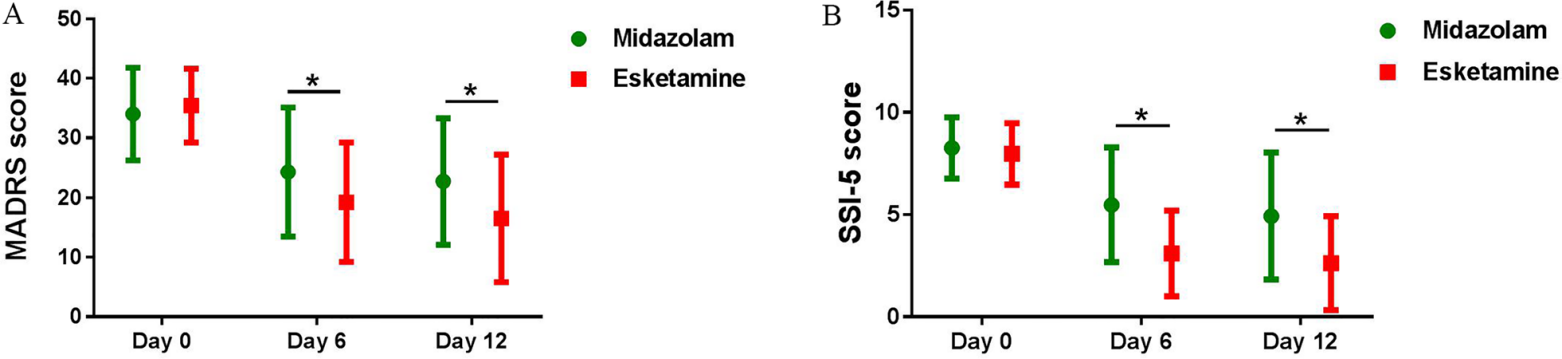
Overall changes in clinical symptoms between groups at Days 0, 6, and 12. **A** A significant decrease in MADRS score in both groups from baseline to Days 6 and 12. **B** A significant decrease in SSI-5 score in both groups from baseline to Days 6 and 12. Asterisk (*) represents significant difference between groups (*P < 0.05). *MADRS* Montgomery–Asberg Depression Rating Scale, *SSI* Beck Scale for Suicide Ideation

The MADRS total score showed a significant time by drug interaction effect (F = 3.345, P = 0.039), time main effect (F = 64.685, P < 0.001), and drug main effect (F = 4.409, P = 0.041) (Additional file 1: Table S1). Post hoc pairwise comparison found the esketamine group had significantly lower MADRS score compared with the midazolam group at Days 6 and 12, and both groups showed a significant decrease in MADRS score from baseline to Days 6 and 12 (Fig. 2A, Additional file 1: Table S2).

The SSI-5 score showed a significant time main effect (F = 71.136, P < 0.001) and drug main effect (F = 5.989, P = 0.018), but no significant time by drug interaction effect (F = 1.816, P = 1.816) (Additional file 1: Table S1). Post hoc pairwise comparison found the esketamine group had significantly lower SSI-5 score compared with the midazolam group at Days 6 and 12, and both groups showed a significant decrease in SSI-5 score from baseline to Days 6 and 12 (Fig. 2B, Additional file 1: Table S2).

### Cognitive performance

**Fig. 3:**
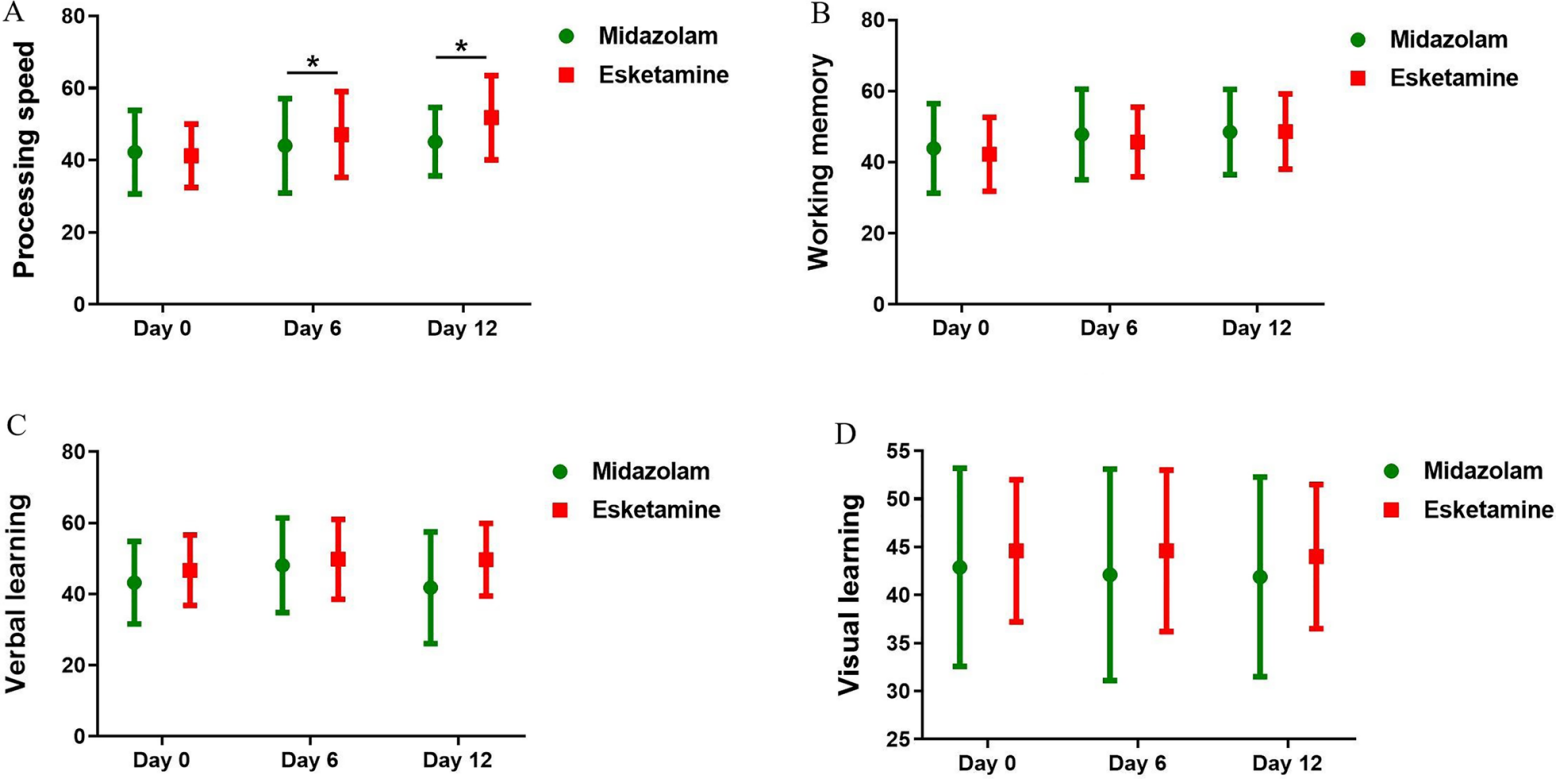
Overall changes in cognitive performance between groups at Days 0, 6, and 12, with significant differences in processing speed between groups at Days 6 and 12. **A** A significant increase in the score of processing speed in esketamine group from baseline to Days 6 and 12. **B** A significant increase in the score of working memory in esketamine group from baseline to Day 12. **C** No significant change of verbal learning in either group from baseline to Day 6 or 12. **D** No significant change of visual learning in either group from baseline to Day 6 or 12. Asterisk (*) represents significant difference between groups (*P < 0.05)

Processing speed showed a significant time main effect (F = 12.803, P < 0.001), drug main effect (F = 6.607, P = 0.013), and interaction effect in time by drug (F = 3.315, P = 0.041) in the linear mixed model (Additional file 1: Table S1). Esketamine group showed significant increases in the mean score of processing speed from baseline to Days 6 and 12, with the effect size 0.566 and 1.032 respectively, suggesting that processing speed had an improvement after esketamine infusions (Additional file 1: Table S2). However, midazolam group did not show any changes in processing speed from baseline to Day 12 (Additional file 1: Table S2). Esketamine group showed significantly better processing speed compared with midazolam group at Days 6 and 12 (Fig. 3A, Additional file 1: Table S2).

Three additional cognitive dimensions, including working memory and verbal learning but not visual learning showed significant time main effect (P < 0.05), but not significant drug or time by drug interaction effect (all P > 0.05, Additional file 1: Table S1). Esketamine group showed an improvement in working memory from baseline to Day 12 in post hoc pairwise comparison (P = 0.006, Additional file 1: Table S2). Both esketamine and midazolam groups did not show any changes in verbal and visual learning from baseline to Day 12(both P > 0.05, Additional file 1: Table S2). Additionally, no significant differences were observed in these three cognitive dimensions between groups (all P > 0.05, Fig. 3B–D, Additional file 1: Table S2).

### Association between baseline cognitive performance and clinical effect over time

The generalized estimation equation showed no significant relationships between baseline cognitive function and anti-depressant response, remission as well as being free of suicidal ideation post-treatment (all P > 0.05). Similar results were also observed in each subgroup sample.

## Discussion

This randomized controlled trial showed that three infusions of esketamine at a subanesthetic dose did not impair cognitive function among depressed adolescents with suicidal ideation. Improvement in processing speed was observed in esketamine-treated patients, and esketamine may be associated with this improvement. Baseline cognitive performance was not associated with esketamine’s antidepressant or antisuicidal effects.

The present findings supported our hypothesis that three-dose of esketamine was safe for cognitive function in adolescents for a 12-day follow-up, while improvement in processing speed was observed, which was consistent with most adult studies. Available studies using single subanesthetic dose ketamine in adults with TRD have not found cognitive impairment in processing speed, working memory, verbal learning and visual learning up to a 14-day follow-up [16–19]. Results from studies with repeated doses of ketamine, such as 4–6 infusions of ketamine over 2–4 weeks also suggest the safety, even some improvement on cognitive function in adults, including visual memory, processing speed, simple working memory, and complex working memory [18, 20, 21, 23, 24]. Up to now only an open-label trial of intranasal ketamine in 12 adolescents with treatment-refractory bipolar depression showed marked improvement in multidimensional symptoms, including mood symptoms, sleep, attention and executive functions [9]. Case report by Dwyer et al. reported a 16-year-old MDD patient with suicidal ideation received seven infusions of ketamine (0.5 mg/kg) over an 8-week hospitalization and standard neurocognitive assessments was evaluated [11]. The detail changes in cognitive function were not reported but mentioned that the patient had returned full-time to school and cognitive assessments had remained stable during outpatient follow-up.

Although the cognitive effect of ketamine in adolescents with depression is less clearly understood, data from pediatric surgery using ketamine as an anaesthetic could be additional evidence to suggest that ketamine does no harm to adolescents’ cognitive function. Ketamine is often used in combination with sleeping pills in order to induce and implement general anesthesia, and can be also used as a supplement of local anesthetics in pediatric anesthesia, with good effect [38]. A controlled study of children aged 9–14 years with lower extremity fractures showed that ultrasound-guided nerve block combined with esketamine showed more postoperative increasing mini-mental state examination (MMSE) score compared with those combined with general anesthesia [39]. An open-label study of adolescents aged 11–19 with chronic pain reported that no significant decline in neurocognitive abilities was observed after low dosage (0.25, 0.5, 1.0, or 1.5 mg/kg/dose three times a day) oral ketamine for 14 days [40]. Instead, a statistically significant improvement in executive functioning and memory was measured at 24–48 h and three months after the last dose of ketamine compared to baseline, along with pain relief.

The available clinical evidences suggested low-dose ketamine administration in adolescents did not lead to significant cognitive impairments, however, animal research showed that low-dose NMDAR antagonists such as ketamine can induce adverse cognitive changes in adolescent rodents [41]. Repeated low-dose (0.1 mg/kg/d for 5 days) NMDAR antagonists administrated to adolescent rats could impair parvalbumin maturation and decrease the number of parvalbumin neurons in the medial prefrontal cortex (PFC), which may result in the neurodevelopmental damage and the potential long-term adverse cognitive changes [42]. In addition, repeated administration of ketamine at low doses (e.g., 5 mg/kg/d for 5 days) to adolescent mice could induce hippocampal CA3 cell death that possibly contributed to lasting cognitive deficits [42]. The PFC and hippocampus play a crucial role in memory processing and executive function. In humans, these brain areas do not fully mature until around age 25, thus prior to maturity they are more sensitive to ketamine-induced cognitive impairments [43]. Although it is possible that the three-dose subanesthetic esketamine infusions paradigm in the present study are conservative enough to preserve normal neurodevelopmental processes in adolescent populations, comprehensive researches need to conduct to examine its long-term cognitive-impairing effects.

Notably, there may be another possible effect, weaker effect on cognitive function induced by low-dose ketamine in developing brains. Drug metabolism is typically faster in adolescents, suggesting adolescents require higher doses of ketamine to achieve antidepressant effects than adults [43]. The 0.25 mg/kg dose administered to adolescents in present study was based on the dosage administrated in adult studies. We suspected that the same ketamine/esketamine dosage would have weaker effects on cognitive function in adolescents compared with adults. In addition, NMDA receptors expression peaks during adolescence and steadily drops-off thereafter, which play a role in learning and memory function. Different concentrations of NMDA receptors between adolescents and adults may result in varying degrees of effects on cognitive function in the context of receiving the same ketamine/esketamine dosage.

Regarding the above-mentioned complex relationships between ketamine dosage and effects on cognitive function in the developing brain, clinical trials evaluating the effects of ketamine across a wide range of doses are need to provide insight on the dosage to strike a balance between antidepressant response and safety.

## Limitations

Several methodological limitations warrant consideration. First, this is a secondary analysis of a randomized trial that was not powered to detect changes in cognition due to its relatively small sample size. However, our sample size met the initial research purpose according to the sample size calculation described detailedly in the trial protocol [31]. It is very important to examine the side effects of esketamine in adolescents, but no studies have explored the impact of repeated infusions of esketamine on adolescents’ cognition. Although the sample size of this study was relatively small, we for the first time gave a full report of the effects of repeated esketamine infusions on cognition of adolescents, which was of great value. A larger sample are needed to validate these findings in the future. Second, cognitive performance was only assessed at baseline, Days 6 and 12, lacking acute (e.g., within hours post-initial infusion) and long-term (e.g., months after treatment) assessments. As mentioned above, slight decreases in delayed recall performance at 40 min after a single infusion were found among adult TRD patients [19]. Ketamine on cognition in different measurement time would likely be different. Second, four dimensions of cognitive function from MCCB were measured in this study, but not including sustained attention, social cognition and other common cognitive impairments in adolescents with depression [44]. Third, suicidal ideation was assessed using the first five items of SSI, but not the entire SSI. Fourth, oral antidepressant medications were provided concomitantly with esketamine or midazolam during the infusion phase, which may create synergies between them as the cause of changes in cognition. Finally, repeated measures of MCCB within 12 days may have learning/practice effects. We have tried to reduce potential learning effect in patients by using different versions (version A, B and C) of visual learning and verbal learning at different timepoints, as described in Method section. However, the other dimensions, processing speed and working memory, have only one version, which might have learning/practice effects. Further studies may be necessary to use a more flexible and comprehensive cognitive battery measuring acute and long-term effect in a larger adolescent sample to validate the present findings.

## Conclusions

Our findings suggested that three low-dose esketamine infusions did not impair the short-term cognitive function of adolescents with MDD and current suicidal ideation; conversely, improvement in processing speed was observed post-infusion. Additional studies are required to clarify the long-term effects of repeated low-dose esketamine infusions on the cognitive function of this population.

### Supplementary Information

**Additional file 1: Table S1.** Results of linear mixed model analysis of clinical symptoms and cognitive performance between groups from baseline to Day 12. **Table S2.** Clinical symptoms and cognitive performance between groups at Days 0, 6, and 12.

## Data Availability

The datasets and analytical code during the current study are available from the corresponding author upon reasonable request.

## Abbreviations

MDD: Major depressive disorder
MCCB: MATRICS Consensus Cognitive Battery
MMSE: Mini-mental state examination
NMDA: *N*-Methyl-d-aspartate
PFC: Prefrontal cortex
TRD: Treatment-resistant depression
SSI: Scale for Suicide Ideation
IV: Intravenous

## References

[1] Berman RM, Cappiello A, Anand A, Oren DA, Heninger GR, Charney DS, Krystal JH (2000). Antidepressant effects of ketamine in depressed patients. Biol Psychiatry.

[2] Murrough JW, Iosifescu DV, Chang LC, Al Jurdi RK, Green CE, Perez AM (2013). Antidepressant efficacy of ketamine in treatment-resistant major depression: a two-site randomized controlled trial. Am J Psychiatry.

[3] Canuso CM, Singh JB, Fedgchin M, Alphs L, Lane R, Lim P (2018). Efficacy and safety of intranasal esketamine for the rapid reduction of symptoms of depression and suicidality in patients at imminent risk for suicide: results of a double-blind, randomized, placebo-controlled study. Am J Psychiatry.

[4] Daly EJ, Singh JB, Fedgchin M, Cooper K, Lim P, Shelton RC (2018). Efficacy and safety of intranasal esketamine adjunctive to oral antidepressant therapy in treatment-resistant depression: a randomized clinical trial. JAMA Psychiat.

[5] Wilkinson ST, Ballard ED, Bloch MH, Mathew SJ, Murrough JW, Feder A (2018). The effect of a single dose of intravenous ketamine on suicidal ideation: a systematic review and individual participant data meta-analysis. Am J Psychiatry.

[6] Zheng W, Zhou YL, Liu WJ, Wang CY, Zhan YN, Li HQ (2018). Rapid and longer-term antidepressant effects of repeated-dose intravenous ketamine for patients with unipolar and bipolar depression. J Psychiatr Res.

[7] Zhan Y, Zhang B, Zhou Y, Zheng W, Liu W, Wang C (2019). A preliminary study of anti-suicidal efficacy of repeated ketamine infusions in depression with suicidal ideation. J Affect Disord.

[8] Dwyer JB, Landeros-Weisenberger A, Johnson JA, Londono Tobon A, Flores JM, Nasir M (2021). Efficacy of intravenous ketamine in adolescent treatment-resistant depression: a randomized midazolam-controlled trial. Am J Psychiatry.

[9] Papolos DF, Teicher MH, Faedda GL, Murphy P, Mattis S (2013). Clinical experience using intranasal ketamine in the treatment of pediatric bipolar disorder/fear of harm phenotype. J Affect Disord.

[10] Cullen KR, Amatya P, Roback MG, Albott CS, Westlund Schreiner M, Ren Y (2018). Intravenous ketamine for adolescents with treatment-resistant depression: an open-label study. J Child Adolesc Psychopharmacol.

[11] Dwyer JB, Beyer C, Wilkinson ST, Ostroff RB, Qayyum Z, Bloch MH (2017). Ketamine as a treatment for adolescent depression: a case report. J Am Acad Child Adolesc Psychiatry.

[12] Weber G, Yao J, Binns S, Namkoong S (2018). Case report of subanesthetic intravenous ketamine infusion for the treatment of neuropathic pain and depression with suicidal features in a pediatric patient. Case Rep Anesthesiol.

[13] Zarrinnegar P, Kothari J, Cheng K (2019). Successful use of ketamine for the treatment of psychotic depression in a teenager. J Child Adolesc Psychopharmacol.

[14] Morgan CJ, Muetzelfeldt L, Curran HV (2010). Consequences of chronic ketamine self-administration upon neurocognitive function and psychological wellbeing: a 1-year longitudinal study. Addiction.

[15] Liang HJ, Lau CG, Tang A, Chan F, Ungvari GS, Tang WK (2013). Cognitive impairments in poly-drug ketamine users. Addict Behav.

[16] Murrough JW, Burdick KE, Levitch CF, Perez AM, Brallier JW, Chang LC (2015). Neurocognitive effects of ketamine and association with antidepressant response in individuals with treatment-resistant depression: a randomized controlled trial. Neuropsychopharmacology.

[17] Chen MH, Li CT, Lin WC, Hong CJ, Tu PC, Bai YM (2018). Cognitive function of patients with treatment-resistant depression after a single low dose of ketamine infusion. J Affect Disord.

[18] Zhou Y, Zheng W, Liu W, Wang C, Zhan Y, Li H (2018). Neurocognitive effects of six ketamine infusions and the association with antidepressant response in patients with unipolar and bipolar depression. J Psychopharmacol.

[19] Murrough JW, Wan LB, Iacoviello B, Collins KA, Solon C, Glicksberg B (2013). Neurocognitive effects of ketamine in treatment-resistant major depression: association with antidepressant response. Psychopharmacology.

[20] Shiroma PR, Thuras P, Wels J, Albott CS, Erbes C, Tye S, Lim KO (2020). Neurocognitive performance of repeated versus single intravenous subanesthetic ketamine in treatment resistant depression. J Affect Disord.

[21] Shiroma PR, Albott CS, Johns B, Thuras P, Wels J, Lim KO (2014). Neurocognitive performance and serial intravenous subanesthetic ketamine in treatment-resistant depression. Int J Neuropsychopharmacol.

[22] Basso L, Bönke L, Aust S, Gärtner M, Heuser-Collier I, Otte C (2020). Antidepressant and neurocognitive effects of serial ketamine administration versus ECT in depressed patients. J Psychiatr Res.

[23] McIntyre RS, Rosenblat JD, Rodrigues NB, Lipsitz O, Chen-Li D, Lee JG (2021). The effect of intravenous ketamine on cognitive functions in adults with treatment-resistant major depressive or bipolar disorders: results from the Canadian rapid treatment center of excellence (CRTCE). Psychiatry Res.

[24] Zhou Y, Wang C, Lan X, Zheng W, Li H, Chao Z (2021). The potential pro-cognitive effects with intravenous subanesthetic ketamine in adults with treatment-resistant major depressive or bipolar disorders and suicidality. J Psychiatr Res.

[25] Zaki N, Chen LN, Lane R, Doherty T, Drevets WC, Morrison RL (2023). Long-term safety and maintenance of response with esketamine nasal spray in participants with treatment-resistant depression: interim results of the SUSTAIN-3 study. Neuropsychopharmacology.

[26] Wajs E, Aluisio L, Holder R, Daly EJ, Lane R, Lim P (2020). Esketamine nasal spray plus oral antidepressant in patients with treatment-resistant depression: assessment of long-term safety in a phase 3, open-label study (SUSTAIN-2). J Clin Psychiatry.

[27] Crisanti C, Enrico P, Fiorentini A, Delvecchio G, Brambilla P (2020). Neurocognitive impact of ketamine treatment in major depressive disorder: a review on human and animal studies. J Affect Disord.

[28] Gill H, Gill B, Rodrigues NB, Lipsitz O, Rosenblat JD, El-Halabi S (2021). The effects of ketamine on cognition in treatment-resistant depression: a systematic review and priority avenues for future research. Neurosci Biobehav Rev.

[29] Ke X, Ding Y, Xu K, He H, Wang D, Deng X (2018). The profile of cognitive impairments in chronic ketamine users. Psychiatry Res.

[30] Zhang C, Xu Y, Zhang B, Hao W, Tang WK (2020). Cognitive impairment in chronic ketamine abusers. Psychiatry Res.

[31] Liu H, Lan X, Wang C, Zhang F, Fu L, Li W (2022). The efficacy and safety of esketamine in the treatment of major depressive disorder with suicidal ideation: study protocol for a randomized controlled trial. BMC Psychiatry.

[32] Montgomery SA, Asberg M (1979). A new depression scale designed to be sensitive to change. Br J Psychiatry.

[33] Beck AT, Kovacs M, Weissman A (1979). Assessment of suicidal intention: the Scale for Suicide Ideation. J Consult Clin Psychol.

[34] Liang S, Xing X, Wang M, Wei D, Tian T, Liu J, Sha S (2021). The MATRICS consensus cognitive battery: psychometric properties of the chinese version in young patients with major depression disorder. Front Psychiatry.

[35] Van Rheenen TE, Rossell SL (2014). An empirical evaluation of the MATRICS consensus cognitive battery in bipolar disorder. Bipolar Disord.

[36] Mohn C, Rund BR (2016). Neurocognitive profile in major depressive disorders: relationship to symptom level and subjective memory complaints. BMC Psychiatry.

[37] Halvorsen M, Waterloo K, Sundet K, Eisemann M, Wang CE (2011). Verbal learning and memory in depression: a 9-year follow-up study. Psychiatry Res.

[38] Zhou Y, Mannan A, Han Y, Liu H, Guan HL, Gao X (2019). Efficacy and safety of prophylactic use of ketamine for prevention of postanesthetic shivering: a systematic review and meta analysis. BMC Anesthesiol.

[39] Wang J, Pu M (2021). Effects of esketamine combined with ultrasound-guided nerve block on cognitive function in children with lower extremity fractures. Am J Transl Res.

[40] Bredlau AL, Harel BT, McDermott MP, Dworkin RH, Korones DN, Dolan JG, Adams HR (2015). Neurocognitive changes after sustained ketamine administration in children with chronic pain. J Palliat Care Med.

[41] Thomases DR, Cass DK, Tseng KY (2013). Periadolescent exposure to the NMDA receptor antagonist MK-801 impairs the functional maturation of local GABAergic circuits in the adult prefrontal cortex. J Neurosci.

[42] Majewski-Tiedeken CR, Rabin CR, Siegel SJ (2008). Ketamine exposure in adult mice leads to increased cell death in C3H, DBA2 and FVB inbred mouse strains. Drug Alcohol Depend.

[43] Tuscher JJ, Luine V, Frankfurt M, Frick KM (2016). Estradiol-mediated spine changes in the dorsal hippocampus and medial prefrontal cortex of ovariectomized female mice depend on ERK and mTOR activation in the dorsal hippocampus. J Neurosci.

[44] Kertz SJ, Petersen DR, Stevens KT (2019). Cognitive and attentional vulnerability to depression in youth: a review. Clin Psychol Rev.

[45] Zhou Y, Lan X, Wang C, Zhang F, Liu H, Fu L, Li W, Ye Y, Hu Z, Chao Z, Ning Y. Effect of repeated intravenous esketamine on adolescents with major depressive disorder and suicidal ideation: a randomized active-placebo-controlled trial. J Am Acad Child Adolesc Psychiatry. 2023. 10.1016/j.jaac.2023.05.031. 10.1016/j.jaac.2023.05.031 37414272

