# Supplementary material for: Short-term cognitive effects of repeated-dose esketamine in adolescents with major depressive disorder and suicidal ideation: a randomized controlled trial
Source: Child Adolesc Psychiatry Ment Health. 2023 Sep 14;17:108. doi: 10.1186/s13034-023-00647-2 (PMC10503003; doi:10.1186/s13034-023-00647-2)
Supplement: Supplementary file 1 — Additional file 1: Table S1. Results of linear mixed model analysis of clinical symptoms and cognitive performance between groups from baseline to Day 12. Table S2. Clinical symptoms and cognitive performance between groups at Days 0, 6, and 12. [file 13034_2023_647_MOESM1_ESM.docx]

Table S1. Results of linear mixed model analysis of clinical symptoms and cognitive performance between groups from baseline to Day 12.

|  | Time main effect | |  | Drug main effect | |  | Time by drug interaction | |
| --- | --- | --- | --- | --- | --- | --- | --- | --- |
|  | F | P |  | F | P |  | F | P |
| MADRS score | 64.685 | <0.001 |  | 4.409 | 0.041 |  | 3.345 | 0.039 |
| SSI-5 score | 71.136 | <0.001 |  | 5.989 | 0.018 |  | 1.816 | 0.168 |
| Processing speed | 12.803 | <0.001 |  | 6.607 | 0.013 |  | 3.315 | 0.041 |
| Working memory | 7.936 | <0.001 |  | 0.001 | 0.976 |  | 0.268 | 0.766 |
| Verbal learning | 4.914 | 0.009 |  | 0.520 | 0.474 |  | 1.551 | 0.217 |
| Visual learning | 0.304 | 0.738 |  | 0.186 | 0.668 |  | 0.109 | 0.897 |

Abbreviations: MADRS= Montgomery-Asberg Depression Rating Scale. SSI= Beck Scale for Suicide Ideation.

Table S2. Clinical symptoms and cognitive performance between groups at Days 0, 6, and 12.

|  | Midazolam | |  | Compared with Day 0 | | |  | Esketamine | |  | Compared with Day 0 | | |  | Midazolam vs Esketamine | | |
| --- | --- | --- | --- | --- | --- | --- | --- | --- | --- | --- | --- | --- | --- | --- | --- | --- | --- |
|  | Mean | SD |  | t | p | Cohen’s d |  | Mean | SD |  | t | p | Cohen’s d |  | t | p | Cohen’s d |
| **MADRS score** |  |  |  |  |  |  |  |  |  |  |  |  |  |  |  |  |  |
| Day 0 | 36.1 | 7.4 |  |  |  |  |  | 35.5 | 6.5 |  |  |  |  |  | 0.214 | 0.831 | -- |
| Day 6 | 27.3 | 11.1 |  | 4.520 | <0.001 | -0.933 |  | 20.6 | 9.5 |  | 7.838 | <0.001 | -1.830 |  | 2.479 | 0.015 | 0.079 |
| Day 12 | 24.8 | 11.8 |  | 5.882 | <0.001 | 1.147 |  | 17.8 | 10.8 |  | 9.268 | <0.001 | -1.975 |  | 2.433 | 0.017 | -0.076 |
| **SSI-5 score** |  |  |  |  |  |  |  |  |  |  |  |  |  |  |  |  |  |
| Day 0 | 8.6 | 1.5 |  |  |  |  |  | 8.1 | 1.4 |  |  |  |  |  | 0.726 | 0.469 | -- |
| Day 6 | 4.8 | 3.2 |  | 6.199 | <0.001 | -1.507 |  | 3.2 | 3.1 |  | 8.128 | <0.001 | -1.994 |  | 2.126 | 0.036 | -6.584 |
| Day 12 | 5.0 | 3.5 |  | 6.013 | <0.001 | -1.380 |  | 2.7 | 3.1 |  | 8.895 | <0.001 | -2.224 |  | 2.781 | 0.006 | -6.743 |
| **Processing speed** | |  |  |  |  |  |  |  |  |  |  |  |  |  |  |  |  |
| Day 0 | 42.2 | 11.6 |  |  |  |  |  | 41.2 | 8.8 |  |  |  |  |  | 0.079 | 0.937 | -- |
| Day 6 | 44.0 | 13.1 |  | -1.010 | 0.947 | -- |  | 47.1 | 11.9 |  | -3.387 | 0.003 | 0.566 |  | -2.041 | 0.043 | 0.516 |
| Day 12 | 45.1 | 9.5 |  | -1.730 | 0.261 | -- |  | 51.8 | 11.7 |  | -5.378 | <0.001 | 1.032 |  | -3.101 | 0.002 | 0.587 |
| **Working memory** | | |  |  |  |  |  |  |  |  |  |  |  |  |  |  |  |
| Day 0 | 43.9 | 12.6 |  |  |  |  |  | 42.2 | 10.4 |  |  |  |  |  | 0.191 | 0.849 | -- |
| Day 6 | 47.8 | 12.8 |  | -1.989 | 0.149 | -- |  | 45.7 | 9.8 |  | -1.849 | 0.203 | -- |  | 0.360 | 0.720 | -- |
| Day 12 | 48.5 | 12.0 |  | -2.304 | 0.070 | -- |  | 48.6 | 10.6 |  | -3.170 | 0.006 | 0.610 |  | -0.572 | 0.569 | -- |
| **Verbal learning** | | |  |  |  |  |  |  |  |  |  |  |  |  |  |  |  |
| Day 0 | 43.2 | 11.6 |  |  |  |  |  | 46.7 | 9.9 |  |  |  |  |  | -0.186 | 0.852 | -- |
| Day 6 | 48.1 | 13.3 |  | -2.343 | 0.064 | -- |  | 49.8 | 11.2 |  | -1.557 | 0.369 | -- |  | 0.591 | 0.555 | -- |
| Day 12 | 41.8 | 15.7 |  | 0.797 | 1.000 | -- |  | 49.7 | 10.2 |  | -0.864 | 1.000 | -- |  | -1.699 | 0.092 | -- |
| **Visual learning** |  |  |  |  |  |  |  |  |  |  |  |  |  |  |  |  |  |
| Day 0 | 42.9 | 10.3 |  |  |  |  |  | 44.6 | 7.4 |  |  |  |  |  | -0.201 | 0.841 | -- |
| Day 6 | 42.1 | 11.0 |  | 0.478 | 1.000 | -- |  | 44.6 | 8.4 |  | 0.000 | 1.000 | -- |  | -0.637 | 0.525 | -- |
| Day 12 | 41.9 | 10.4 |  | 0.465 | 1.000 | -- |  | 44.0 | 7.5 |  | 0.638 | 1.000 | -- |  | -0.047 | 0.963 | -- |

Abbreviations: MADRS= Montgomery-Asberg Depression Rating Scale. SSI= Beck Scale for Suicide Ideation.
